# Headspace Solid Phase Micro-Extraction of Volatile Constituents Produced from Saudi *Ruta chalepensis* and Molecular Docking Study of Potential Antioxidant Activity

**DOI:** 10.3390/molecules28041891

**Published:** 2023-02-16

**Authors:** Hanan Y. Aati, Hala Attia, Razan Babtin, Najla Al-Qahtani, Juergen Wanner

**Affiliations:** 1 Department of Pharmacognosy, College of Pharmacy, King Saud University, P.O. Box 2457, Riyadh 11451, Saudi Arabia; 2 Department of Pharmacology and Toxicology, College of Pharmacy, King Saud University, P.O. Box 2457, Riyadh 11451, Saudi Arabia; 3 College of Pharmacy, King Saud University, P.O. Box 2457, Riyadh 11451, Saudi Arabia; 4 Kurt Kitzing Co., Hinterm Alten Schloss 21, D-86757 Wallerstein, Germany

**Keywords:** *Ruta chalepensis*, essential oil, HS-SPAM, antioxidant, in silico, molecular docking

## Abstract

*Ruta chalepensis* L., commonly known as Shazab in Saudi Arabia, is one of the famous culinary plants belonging to the Rutaceae family. It is commonly used in ethnomedicine in treating numerous diseases. This study was performed to characterize the essential oil isolated from Saudi species using a relatively new advanced headspace solid-phase microextraction technique. Following that, the antioxidant activity of the extracted oil was assessed using in vitro techniques such as the DPPH and nitric oxide scavenging tests, as well as the reducing power FRAP study and the molecular docking tool. The essential oil yield of the dried plant was 0.83% (*v*/*w*). Gas chromatography joined with a mass spectrometer was used to determine the chemical composition of the pale-yellow essential oil. Sixty-eight constituents were detected, representing 97.70% of the total oil content. The major constituents were aliphatic ketones dominated by 2-undecanone (37.30%) and 2-nonanone (20.00%), with minor constituents of mono and sesquiterpenoids chemical classes. Nicotinamide adenine dinucleotide phosphate (NADPH) oxidase is one of the major causes of many contemporary diseases due to its ability to create a reactive oxygen species (ROS). Thus, molecular docking was used to confirm that some oil phytoconstituents have good docking scores compared to the standard antioxidant drug (Vitamin C), indicating great binding compatibility between the (NADPH) oxidase receptor site and the ligand. In conclusion, our findings suggest that the oil could be used safely and as a cost-effective remedy in treating various modern diseases caused by free radical formation.

## 1. Introduction

Nowadays, the rapid development and modern way of life worldwide, which is characterized by behaviors that support external factors, has led to the spread of many incurable diseases such as cardiovascular diseases, central nervous system problems, and cancer [1]. All these diseases are related to the presence of a large amount of free degenerative radicals inside the body, consequently causing the destruction of the body cells (oxidative stress) [2]. Hence, antioxidant agents that scavenge these free radicals and limit their destructive effects on the body cells remain the only solution. There are many antioxidant agents; however, antioxidants of natural origin are the best and safest way to combat and eliminate free radicals without harming the normal body cells [3]. Therefore, since ancient times, plants have been a significant source of effective compounds that act as cell guards to eliminate free radicals without harming other body cells. Extensive research has recently revealed the efficacy of many medicinal herbs in treating various modern diseases caused by free radicals [4]. Medicinal herbs contain many secondary metabolites, such as flavonoids, alkaloids, terpenes, and other phenolic compounds that work individually or synergistically to protect cells from harmful external factors. This leads to an increase in their lifespan and restoration of vitality [5].

Aromatic medicinal plants have biological properties that could be useful in the cosmetic, medical, and pharmaceutical fields [6]. Rutaceae, an essential plant family, is considered a source of a wide variety of secondary metabolites with antioxidant, antibacterial, antifungal, antihelmintic, spasmolytic, antitumor, antidepressant, analgesic, and anti-inflammatory activities [7]. The common Ruta species studied for their oil constituents and biological activities are *R. graveolens, R. chalepensis*, and *R. montana* [8].

*Ruta chalepensis* L., commonly known as Shazab in Saudi Arabia, is a well-known member of the Rutaceae family and is commonly used in folk medicine to treat mental disorders, rheumatism, dropsy, neuralgia, fever, bleeding and menstrual issues, nervous disorders, and convulsions [9]. It has a strong aromatic odor and numerous biological activities, which could be attributed to the presence of different secondary metabolite classes such as saponins, flavonoids, triterpenes, and essential oils, additional to coumarins, phenols, and alkaloids [10].

The most important secondary metabolites in recent years have been essential oils extracted from natural sources, containing hydrocarbon compounds with hydrogenated, oxygenated, and dehydrogenated functional groups [11]. Most of these constituents are odorous mono or sesquiterpenoids found in various parts of the plant that evaporate at normal temperatures [12]. Many techniques are used to extract essential oil from aromatic plants, and the volume, percentage concentration, and many identified constituents vary. The headspace solid-phase microextraction (HS-SPME) technique is a relatively new advanced technique for volatile oil isolation. HS-SPME was constructed to isolate volatile constituents with a wide range of boiling points without forming artifacts, and the harmful organic solvents are not involved in the process [13].

Molecular docking is a time-efficient and cost-effective method used to explore the complexity of protein–ligand interactions, which helps design, synthesize, and discover therapeutically important drugs. It is used in protein engineering and medicinal chemistry as well as other biological and medicinal areas [14]. Many enzyme systems such as NADPH oxidase, cytochrome P450 reductase, nitric oxide synthases, and xanthine oxidase when inhibited will exhibit antioxidant and reactive oxygen scavenging potencies. To study the ability of the oil to inhibit NADPH oxidase enzymes, all compounds released from the GC-MS analysis were assessed, along with ascorbic acid, and docked against the NADPH oxidase enzyme.

Few previous studies have evaluated the antioxidant potency of the titled plant essential oil, but this study showed the first work carried out to identify the chemical compositions of the oil isolated from aerial parts of Saudi species using the headspace solid-phase microextraction (HS-SPME) method and screening its antioxidant potential using in vitro and in silico studies on the most active compounds. This study aimed to determine a safe and cost-effective remedy that can be used as a natural antioxidant to combat many incurable diseases, either alone or in combination with other synthetic antioxidant drugs.

## 2. Results

### 2.1. Chemical Composition of Essential Oil

**Figure 1:**
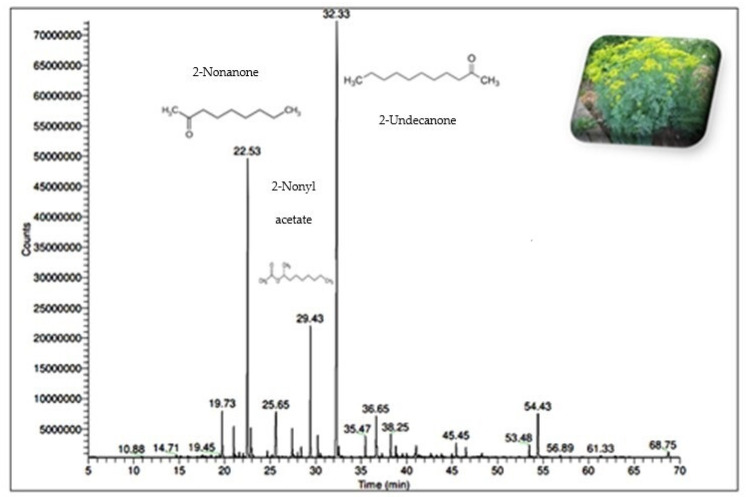
GC-MS chromatogram for *Ruta chalepensis* essential oil compositions. Main components were detected at Rts 22.53, 29.43, and 32.33 and were assigned for 2-Nonanone, 2-Nonyl acetate, and 2-Undecanone, respectively.

The chemical complexity of the essential oil obtained by the HS-SPME of the dry plant powder aerial part was determined using GC–MS analysis and is tabulated in Table 1, Figure 1. Compounds are listed in the order of elution on the capillary column, with retention times (Rts).

The application of headspace solid-phase microextraction (HS-SPME) to the aerial part of the Saudi chalepensis species resulted in 0.83% (*v/w*) yields of pale-yellow essential oil. The volatile constituents of the essential oil were identified by comparing their mass spectra to the NIST08 mass spectral database.

The relative percentages of the individual components were calculated based on the GC/MS peak area. The GC/MS analysis of the *R. chalepensis* oil revealed the presence of 68 aroma constituents with a total yield of 97.70%. This showed that this essential oil predominantly contains aliphatic ketones and some minor aliphatic alcohols; however, they generally lack terpene components. Our findings revealed that the long-chain aliphatic ketones are the class of the most abundant component and account for approximately half of the total oil content (63.80%). 2-Undecanone represented 37.30% of total oil yield and, followed by 2-nonanone (20.00%), was a major compound. Camphor and limonene (2.70% and 2.60%, respectively) dominated the monoterpenes and occupied 9.90% of the total oil yield. Sesquiterpenes and diterpenes were the other terpenoid chemical classes identified in the oil extracted from the selected plant growing in Saudi Arabia, with percentages of 3.50% and 0.10%, respectively.

### 2.2. In Vitro Antioxidant Study

The DPPH (2,2-diphenyl–1-picryhydrazyl) and nitric oxide scavenging studies, as well as reducing power (FRAP) assays, were used to evaluate the obtained oil antioxidant potency (Table 2 and Table 3).

### 2.3. In-Silico Study for the Phytoconstituents in Essential Oil

**Figure 2:**
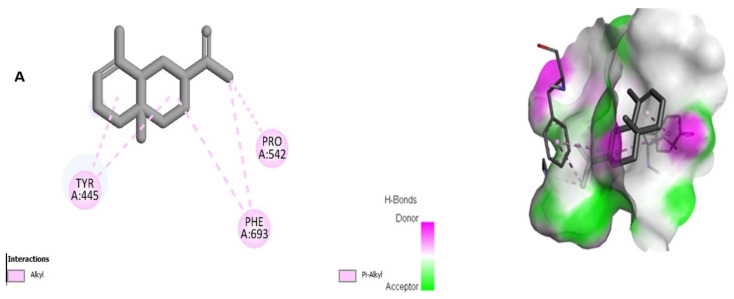

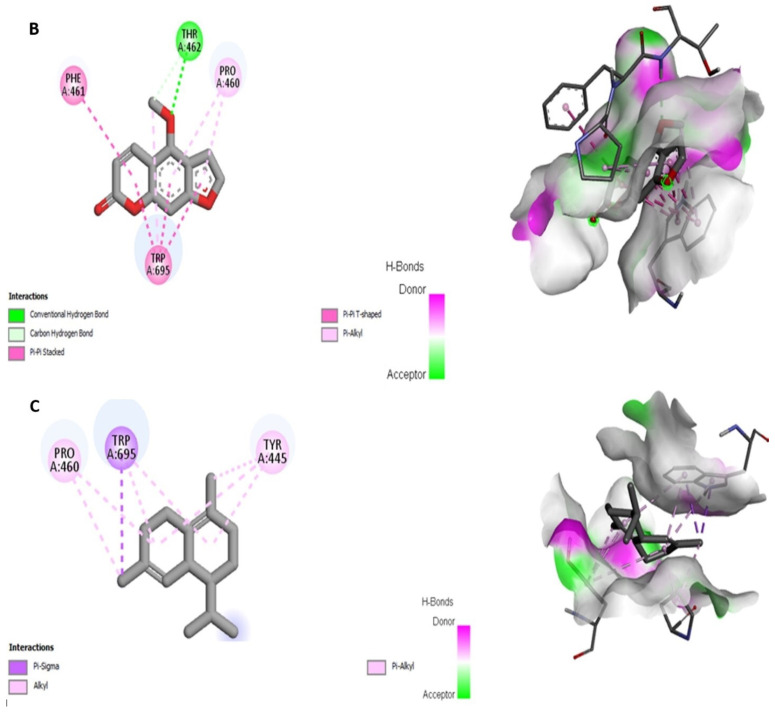

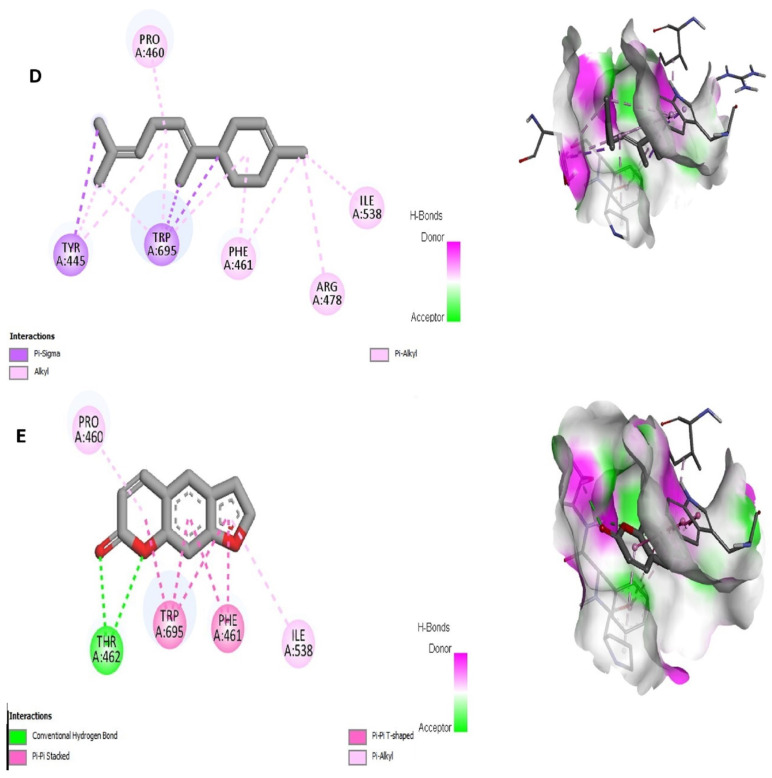

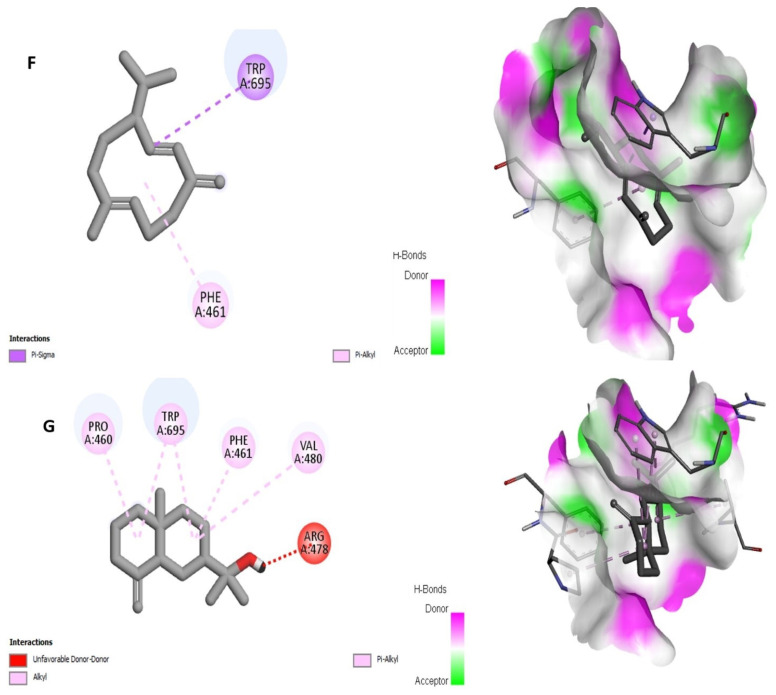
The 2D and 3D interactions of (**A**) “*α*-Selinene”, (**B**) “Bergapten”, (**C**) “*δ*-Cadinene”, (**D**) “(*E*)-*α*-bisabolene”, (**E**) “Psoralen”, (**F**) “Germacrene D”, and (**G**) *“β*-Eudesmol with the receptor NADPH oxidase.

To determine whether *R. chalepensis* oil is responsible for antioxidant activity, all compounds from the GC/MS analysis and ascorbic acid (standard) were docked against the NADPH enzyme. The hydrophobic interactions, such as pi-alkyl and alkyl in addition to hydrogen bonds, play critical roles in protein–ligand interactions and support its stability. Overall, psoralen, (*E*)-*α*-bisabolene, *δ*-cadinene, bergapten, germacrene D, *β*-eudesmol, and α-selinene may have some potential as inhibitors of important enzyme NADPH Oxidase and may have contributed to the antioxidant properties of *R. chalepensis* oil, either singly or synergistically. Table 4 shows the predicted docking score outcomes and the interactions between proteins and ligands; the two- and three-dimensional structures are available in Figure 2.

### 2.4. Acute Systemic Toxicity (LD_50_)

In this study, the acute systemic toxicity of the titled plant essential oil was assessed via the oral route. No signs of toxicity and mortality were recorded at a dosage range between 500 and 2000 mg/kg. The animals were observed to detect any general behavioral changes and other characteristics such as respiration, body weight, temperature, food and water intake, diarrhea, urination, drowsiness, sedation, tremor, change in skin, hair and eye color, as well as coma.

## 3. Discussion

Saudi Arabia has a distinct climate and geographical location, with many mountains, a desert, and plains resulting in diverse plant biodiversity. According to the literature review, 309 genera with 471 species in 89 families are used in ethnomedicine and to treat various modern diseases [15]. *Ruta* species are well-known aromatic plants, and their isolated oils provide significant aromaticity. Essential oils are a complex mixture of different constituent classes such as aliphatic alcohols, monoterpenes, sesquiterpenes, ketones, aldehydes, and acids [8]. The current study shows the isolation of pale-yellow essential oil with a strong and penetrating odor. The identification of the chemical constituents of isolated oils using the GC/MS chromatographic method revealed 68 volatile organic constituents, and they accounted for 97.70% of their essence (Table 1). Long-chain aliphatic ketones accounted for 63.80% of the constituents, with 2-undecanone being the most abundant (37.30%) and 2-nonanone accounting for the remaining 20.00%. Monoterpenes cyclic and hydrocarbons were found in reasonable amounts in the air-dried aerial part of the selected plant growing in Saudi Arabia, with camphor (2.70%), limonene (2.60%), and carvone (1.30%) as major constituents. This chemical class of volatile matter is responsible for many biological activities and as the fragrances and odor sensations of many plants [16]. In addition to monoterpenes, the sesquiterpene was present in a volatile fraction at 3.50%, with (*E*)-*β*-caryophyllene (0.70%) predominating followed by 0.3% of *α*-curcumin and *β*-sesquiphellandrene. Diterpene was present in trace amounts and was dominated by moskachane D. Other chemical constituents represented 20.40% of the oil content, including fatty acids, alcohols, and phenolic compounds (see Table 1).

Previous studies revealed that the main composition of the oil produced by the *R. chalepensis* species growing in Argentina, Turkey, Iran, and India was 2-undecanone (38.1, 66.5, 52.5, and 41.3–67.8, respectively). In contrast, 2-nonanone was a significant constituent in Turkish, Italian, Iranian, and Indian oils (16.2, 49.9, 24.1, and 5.2–33.6%, respectively). The major components of the Saudi oil isolated from plants were 2-undecanone, 2-tridecanone, elemol, and *β*-eudesmol (16.2, 49.9, 24.1, and 5.2–33.6%, respectively) [8]. The key components of the Greek oil were 2-methyl octyl acetate (44.0%) and *β*-phellandrine (10.7%). These variations in phytochemical components may be due to the different geographical locations, climates, and weather conditions under which the plant was collected [17]. 2-Undecanone is the main constituent of the oils extracted from *Ruta* species; therefore, it has been proposed as a suitable chemotaxonomic marker for the genus *Ruta*. However, 2-nonanone could be another marker compound for the chemotaxonomic study of this genus [8].

Hydrodistillation, steam distillation, supercritical fluid extraction, and headspace solid-phase microextraction (HS-SPME) are all methods for extracting essential oil. HS-SPME is a relatively new advanced technique for the isolation of volatile constituents. It extracts organic compounds from its matrix using a fine quartz fiber with a polymeric coating. Then, it directly transfers them into the injector of a gas chromatograph for thermal desorption and analysis. It was designed to extract volatile compounds with a wide range of boiling points without forming artifacts. Additionally, it is a rapid method that does not require solvents or a huge number of instruments. Further, it shields the oil constituents and provides more components than other volatile extraction techniques. This study was the first report written on the essential oils extracted from Saudi *R. chalepensis* aerial parts using HS-SPME, and it analyzed its constituent using GC/MS.

In recent years, there has been a demand to increase the research and development of new medicines derived from natural products. Essential oils and their constituents are categorized as safe secondary metabolites isolated from natural products. They have been observed with numerous biological activities [8]. The volatile constituents isolated from various genus of *Ruta* used in folk medicine remedies and the literature have been studied their biological activity. Insecticidal, anti-inflammatory, antioxidant, antiprotozoal, antimicrobial, cytotoxic, herbicidal, larvicidal, and anthelmintic are among the important biological activities of *Ruta* oils [8].

The spread of many modern diseases, as a result of the presence of free radical stimuli these days, requires counteracting agents known scientifically as antioxidants. This is very important, especially with those derived from new, safe, and effective natural products. Various studies confirmed the antioxidant potency of oils [18,19], and it depends on their constituents. Many essential oils demonstrated significant antioxidant activity and referred to the presence of oxygenated monoterpenes such as alcohols, ketons, aldehydes, and esters.

In this work, three in vitro studies were used to evaluate the antioxidant potency of essential oil extracted from the aerial part of *R. chalepensis* Saudi species. The results of DPPH, nitric oxide scavenging, and FRAP reducing power studies showed a concentration-dependent antiradical potency when compared to a strong natural antioxidant drug, ascorbic acid. An oil dose of 100 µL gave 86.25 ± 1.22, 82.30 ± 4.73, and 1.43 ± 0.05 compared to ascorbic acid, which gave 91.07 ± 2.28, 89.68 ± 3.49, and 1.97 ± 0.11, respectively (Table 2). Regarding the IC50% inhibition of free radical formation, values were determined as 40.26%, 36.88%, and 19.09% in comparison to the potent antioxidant control drug ascorbic acid, which was 20.94%, 24.75%, and 16.10%, respectively, using DPPH radical inhibition, nitric oxide scavenging, and FRAP reducing power assessment studies, respectively (Table 3). Therefore, it is obvious that the effectiveness of the isolated oil by HS-SPME was proven as a natural and safe source. Further, its antioxidant effect increases with increasing doses, and the oil is considered safe even when high doses are used up to 2000 mg/kg.

Recently, there has been an increase in the use of computational methods, such as molecular docking, to assess the interactions between natural products and their biological targets [20,21]. The ROS are produced in the mitochondria via oxidative phosphorylation and NADPH oxidase. Additionally, NADPH oxidase facilitates abnormal cell proliferation and intracellular signaling processes. This has been detected in numerous modern diseases; thus, when inhibited, it exhibits antioxidant and reactive oxygen scavenging potencies [22]. To understand the ability of the oil to inhibit NADPH oxidase enzymes and to find a correlation between the antioxidant results, all compounds obtained from the GC-MS analysis, along with ascorbic acid (standard), were docked against the NADPH oxidase enzyme. The hydrophobic interactions, such as pi-alkyl and alkyl as well as hydrogen bonds, play a significant role in protein–ligand interactions, and they support its stability. Overall, psoralen, (*E*)-*α*-bisabolene, *δ*-cadinene, bergapten, germacrene D, *β*-eudesmol, and α-Selinene may have some potential as inhibitors of important oxidative enzyme NADPH and may contribute either singly or in synergistically to achieve the antioxidant properties of the titled plant essential oil.

## 4. Materials and Methods

### 4.1. Plant Material, Essential Oil Extraction, and GC/MS Analysis

A half kilogram of fresh plant aerial parts was collected during the flowering stage in May 2021 from the Fayfa mountains in Jizan province, Kingdom of Saudi Arabia. The plants were exposed to cold air-drying, the drying continued until the moisture content of the aerial parts were reduced to about 10% (spent 10 days), which is low enough to keep plants secure from microbial growth, and the drying ratio was (500:150). The plant sample was identified by taxonomist Dr. Rajakrishnan Rajagopal, King Saud University. The specimen was deposited in the Herbarium, KSU (Voucher#KSU20278); all protocols involving plants adhered to relevant ethical guidelines/regulations of KSU.

The HS-SPME, GC/MS, and identification of secondary metabolites were performed according to the literature described in Aati et al. [23]. The powder of the plant was located in a headspace vial (5 mL). Then, it was supplemented on an SPME fiber (PDMS/DVB/Carboxen, SUPELCO part no. 57298-U) for 1 h at 80 °C in a metal block in such a way that the plant material was subjected to the elevated temperature, while the SPME fiber was kept cold (room temperature). At the GC injector site, the supplemented fiber was placed in it, and the fiber desorbed for 1 min at 250 °C.

GC–MS analyses were performed using Thermo Fisher Scientific Trace GC Ultra, and they used a split/split less injector heated at 250 °C and connected to a 50 m × 0.25 mm × 1.0 μm SE-52 (95% polydimethylsiloxane, 5% polydiphenylsiloxane) capillary column. The essential oil component was characterized using a Thermo Fisher Scientific ISQ mass spectrometer connected with a GC/MS interface heated at 250 °C and used the electron ionization mode at 70 eV and a filament at 50 μA. Furthermore, a scan range of 40–500 amu was used, and an ion source ran at 230 °C. The oven heated for 1 min at 60 °C, and then heating increased to 230 °C at a rate of 3 °C/min and a 230 °C isotherm for 12.3 min (gradient program). Helium was used as carrier gas and flowed at a constant rate of 1.5 mL/min.

The volatile constituents of the essential oil were identified by comparing their mass spectra to NIST08 mass spectral database. The relative percentages of the individual components were calculated based on the GC/MS peak area.

### 4.2. Biological Activity

#### Antioxidant Activity

*Ruta chalepensis* oil antioxidant activities included determining reducing power and free radical scavenging potencies. All antioxidant activities were measured using ascorbic acid as the standard. The DPPH, nitric oxide radical scavenging assays, and FRAP activities were performed according to the literature, with minor modifications [12].

- DPPH Assay

A 90 µL (0.1 mM) DPPH solution was prepared and added to a 96 well plate, followed by 10 µL of the oil being studied. The absorbance was read at 517 nm using a BioTek Synergy HT microplate reader, after incubating for 30 min [12].

- Nitric oxide radical scavenging assay

The Griess reaction was used to measure the nitrite ions produced when sodium nitroprusside interacted with oxygen. Green et al. described the use of this assay [24]; 10 mM sodium nitroprusside (3.0 mL) in phosphate-buffered saline (pH 7.4) and oil concentrations of (20–100 μg/mL) were used in the reaction. The resulting solution was then incubated for 60 min at 25 °C. Next, 5.0 mL of Griess reagent (1% sulphanilamide, 0.1% NEDD in 2% H_3_PO_4_) was added to the incubated sample, and the absorbance of the formed chromophore was measured at 546 nm against a reagent blank. The percentage inhibition of the nitrite ions generated was observed. For comparison, standard ascorbic acid was used.

- FRAP Assay

A total of 100 µL of oil (1 mg/mL) was mixed with FRAP reagent (2 mL), and the mixture was incubated for 30 min. A BioTek Synergy HT microplate reader was used to measure the absorbance at 593 nm. Similarly, the blank sample was prepared without oil [12].

### 4.3. In Silico Activities

#### Molecular Dockings

The receptor nicotinamide adenine dinucleotide phosphate (NADPH) oxidase was obtained from the RCSB Protein Data Bank (https://www.rcsb.org/, accessed on 30 May 2022). Using the Discovery studio, the proteins were first prepared for docking by removing water and ligands, inserting polar hydrogen atoms, and saving them in PDB format. Then, the ligand in SDF format from PubChem (https://pubchem.ncbi.nlm.nih.gov, accessed on 30 May 2022) was downloaded. All the compounds tentatively identified in the GC-MS were docked, and the best docked compounds in terms of best binding affinity were presented in the results. In the preparatory steps of compounds to ligands, these were converted to pdbqt format and then were converted to ligands using the Open Babel. The prepared and optimized ligands were docked blindly in the protein’s grid box to allow them to find any suitable binding location with PyRx [25]. The docking studies were validated by superimposing the co-crystallized ligand (Ascorbic acid) with extracted ascorbic acid from the crystal structure and redocking it to the crystal structures of NADPH Oxidase. The Ligplot software was used to visualize 2D structures of ligand–protein interactions [26].

### 4.4. Acute Systemic Toxicity (LD_50_)

Karber et al. described the method of determining the LD_50_ [27]. The experiment underwent the ethics reference number: KSU-SE–22–71.

### 4.5. Statistical Analysis

Results were expressed as mean ± SD and evaluated using one way ANOVA followed by a post hoc Tukey test to compare the control and treatment groups. Differences were considered statistically significant at *p* < 0.05, and SPSS software (v.11.5, IBM, Armonk, NY, USA) was used to perform all statistical analyses.

## 5. Conclusions

Free radicals lead to numerous modern diseases. Phytoconstituents derived from natural products were revealed to possess antioxidant activity with a safe and effective profile. This study on the Saudi species of *R. chalepensis* oil revealed 68 compounds identified in the oil for the first time using HS-SPME and GC/MS techniques. The essential oil studied was governed by two aliphatic ketones, 2-undecanone and 2-nonanone, and contained a lesser proportion of terpenes. This prompted us to investigate the antioxidant properties of oil phytoconstituents using molecular docking. The molecular docking findings revealed that psoralen, (*E*)-*α*-bisabolene, *δ*-cadinene, bergapten, germacrene D, *β*-eudesmol, and α-Selinene had good docking scores. The findings support the approved traditional use of the titled plant as a folk remedy for scavenging free radicals. Finally, the *R. chalepensis* essential oil has the ability to scavenge free radicals. Therefore, it could be used to treat chronic or degenerative diseases and to determine non-toxic and selective inhibitors of NADPH oxidases, delivering innovative drugs for treating diseases caused by oxidative stress.

## Figures and Tables

**Table 1:** Composition of the *Ruta chalepensis* aerial part essential oil extracted using HS-SPME.

#	Compound	Rt (min.)	RI	%Conc.
1	Ethyl 2-methylbutyrate	10.88	845.00	0.10
2	*α*-Thujene	14.71	931.00	0.10
3	*α*-Pinene	15.15	940.00	0.10
4	Camphene	15.94	957.00	0.10
5	Benzaldehyde	16.16	961.00	0.10
6	Sabinene	17.00	979.00	0.10
7	*β*-Pinene	17.30	985.00	0.10
8	2-Octanone	17.40	987.00	0.10
9	Myrcene	17.58	991.00	0.10
10	Octan–2-ol	17.85	996.00	0.10
11	*α*-Phellandrene	18.51	1010.00	0.10
12	*α*-Terpinene	19.11	1022.00	0.10
13	*p*-Cymene	19.45	1029.00	0.20
14	Limonene	19.73	1034.00	2.60
15	Artemisia ketone	21.00	1060.00	1.60
16	*γ*-Terpinene	21.17	1063.00	0.10
17	*cis*-sabinene hydrate	21.61	1072.00	0.30
18	2-Nonanone	22.53	1090.00	20.00
19	Terpinen–4-ol	22.70	1094.00	0.20
20	2-Nonanol	22.87	1097.00	1.50
21	Linalool	22.97	1099.00	0.50
22	Nonanal	23.10	1102.00	0.10
23	Oct–2-yl acetate	24.69	1134.00	0.30
24	Geijerene	25.59	1153.00	1.40
25	Camphor	25.65	1154.00	2.70
26	Borneol	26.70	1176.00	0.10
27	2-Decanone	27.43	1190.00	1.60
28	*α*-Terpineol	27.74	1197.00	0.10
29	*cis*-dihydrocarvone	28.02	1203.00	0.30
30	*trans*-dihydrocarvone	28.42	1211.00	0.60
31	2-Nonyl acetate	29.43	1233.00	7.50
32	Carvone	30.24	1250.00	1.30
33	Isogeijerene C	31.00	1266.00	0.10
34	2-Undecanone	32.33	1295.00	37.30
35	2-Undecanol	32.53	1299.00	0.70
36	3-Nonyl acetate	32.87	1306.00	0.10
37	2-Decyl acetate	33.94	1330.00	0.10
38	Piperitenone	34.85	1351.00	0.10
39	6-Dodecanone	35.47	1365.00	1.20
40	(*Z*)-ethyl cinnamate	36.09	1379.00	0.10
41	8-epi-Dictamnol	36.48	1387.00	0.10
42	Biphenyl	36.65	1391.00	2.40
43	2-Dodecanone	36.74	1393.00	0.50
44	*β*-Elemene	37.28	1406.00	0.30
45	2-Undecyl acetate	38.25	1428.00	1.40
46	(E)-*β*-caryophyllene	38.80	1442.00	0.70
47	Dictamnol	38.96	1445.00	0.20
48	(*E*)-*β-*farnesene	39.56	1460.00	0.30
49	(*E*)-Ehyl cinnamate	40.01	1470.00	0.30
50	*α-*curcumene	40.91	1492.00	0.30
51	2-Tridecanone	41.04	1495.00	0.70
52	Germacrene D	41.33	1502.00	0.20
53	*β*-Selinene	41.63	1509.00	0.10
54	*α*-Selinene	41.95	1517.00	0.10
55	*β*-Sesquiphellandrene	42.68	1535.00	0.30
56	*δ*-Cadinene	42.81	1539.00	0.10
57	(*E*)-*α*-Bisabolene	43.31	1551.00	0.20
58	Elemol	43.83	1564.00	0.20
59	(*E*)-Nerolidol	43.99	1568.00	0.10
60	*cis*-Davanone	44.99	1593.00	0.20
61	Dulcinyl	45.45	1605.00	0.80
62	Dill apiole	46.52	1633.00	0.60
63	*β*-Eudesmol	48.13	1675.00	0.10
64	7-Methoxycoumarin	50.60	1742.00	0.10
65	Psoralen	54.43	1851.00	3.20
66	Methyl palmitate	56.89	1923.00	0.10
67	Moskachane D	61.33	2043.00	0.10
68	Bergaptene	62.91	2081.00	0.10
Cyclic Monoterpenes				9.30
Acyclic (Hydrocarbon) Monoterpenes				0.60
Cyclic Sesquiterpenes				3.10
Acyclic (Hydrocarbon) Sesquiterpenes				0.40
Cyclic Diterpenes				0.10
Other Chemical Classes				84.20
Total %				97.70

“Rt” retention time, “RI” retention index, “%Conc” % concentration of the compound described by its peak area.

**Table 2:** Results of the antioxidant activity test for *Ruta chalepensis* oil using DPPH, nitric oxide scavenging, and reducing power (FRAP) assays.

Sample	Dose (µL)	DPPH Scavenging Activity	Nitric oxide Scavenging Activity	Reducing Power (FRAP) Potential
Essential oil	10	11.07 ± 4.68	15.70 ± 12.81	0.32 ± 0.02
Essential oil	20	20.09 ± 6.24	37.57 ± 3.43	0.65 ± 0.07
Essential oil	50	49.67 ± 6.25	49.31 ± 2.76	0.89 ± 0.07
Essential oil	100	86.25 ± 1.22	82.30 ± 4.73	1.43 ± 0.05
Ascorbic acid	100	91.07 ± 2.28	89.68 ± 3.49	1.97 ± 0.11

Values are given as (mean ± SD).

**Table 3:** The antioxidant half-maximal inhibitory concentration IC_50_ inhibition values for the oil.

IC _50_ (% Inhibition)	DPPH Scavenging Method	Nitric Oxide Scavenging Method	Reducing Power (FRAP) Method
Essential oil	40.26%	36.88%	19.09%
Ascorbic acid	20.94%	24.75%	16.10%

**Table 4:** Docking score of phytoconstituents at the active site of the receptor NADPH Oxidase.

Sr. No.	Ligands	Highest to Lowest Mode of Conformation with Corresponding Binding Affinities in ΔG (Kcal/mol)								
		1	2	3	4	5	6	7	8	9
1	7-Methoxycoumarin	−7.6	−7.6	−7.5	−7.4	−7.2	−6.9	−6.9	−6.8	−6.6
2	*α*-Selinene	−7.9	−7.8	−7.6	−7.3	−7.0	−6.7	−6.7	−6.3	−6.3
3	Bergapten	−8.1	−8	−7.9	−7.7	−7.7	−7.6	−7.4	−7.0	−6.9
4	*δ*-Cadinene	−8.3	−8.1	−8.0	−7.9	−7.8	−7.6	−7.5	−7.3	−7.0
5	*β*-Selinene	−7.4	−7.3	−6.9	−6.9	−6.6	−6.2	−6.1	−6.1	−6.0
6	Sesquiphellandrene	−7.6	−7.5	−7.3	−7.2	−7.2	−7.1	−7.1	−6.9	−6.8
7	trans-*β*-Farnesene	−7.7	−7.5	−7.4	−7.3	−7.3	−7.2	−7.2	−7.2	−7.0
8	Nerolidol	−7.5	−7.2	−7.1	−7.1	−6.9	−6.7	−6.7	−6.7	−6.5
9	(*E*)-*α*-bisabolene	−8.5	−8.2	−8.1	−8.0	−7.5	−7.3	−7.2	−7.2	−7.1
10	Psoralen	−8.6	−8.5	−7.9	−7.8	−7.7	−7.6	−7.6	−7.4	−7.4
11	Germacrene D	−8.1	−8	−6.9	−6.9	−6.7	−6.7	−6.6	−6.3	−6.1
12	Biphenyl	−7.8	−7.8	−7.8	−7.8	−7.8	−7.7	−7.7	−7.5	−7.4
13	*β*-Eudesmol	−8.0	−7.8	−7.6	−7.4	−7.3	−6.9	−6.5	−6.4	−6.4
14	Dictamnol	−7.4	−7.1	−6.9	−6.6	−6.5	−6.4	−6.3	−6.2	−6.2
15	Ascorbic acid *(Standard)*	−7.1	−6.9	−6.7	−6.5	−6.4	−6.3	−6.2	−6.1	−6.0

## Data Availability

Not applicable.

## Sample Availability

Samples of the oil are available from the authors.
